# Cognitive deficits caused by a disease-mutation in the α_3_ Na^+^/K^+^-ATPase isoform

**DOI:** 10.1038/srep31972

**Published:** 2016-08-23

**Authors:** Thomas Hellesøe Holm, Toke Jost Isaksen, Simon Glerup, Anders Heuck, Pernille Bøttger, Ernst-Martin Füchtbauer, Steen Nedergaard, Jens Randel Nyengaard, Mogens Andreasen, Poul Nissen, Karin Lykke-Hartmann

**Affiliations:** 1 Aarhus University, Department of Biomedicine, DK-8000 Aarhus, Denmark; 2 Centre for Membrane Pumps in Cells and Disease-PUMPKIN, Danish National Research Foundation, Aarhus University, Department of Molecular Biology and Genetics, DK-8000 Aarhus C, Denmark; 3 Aarhus University, Department of Molecular Biology and Genetics, DK-8000 Aarhus, Denmark; 4 Stereology and Electron Microscopy Laboratory, Center for Stochastic Geometry and Advanced Bioimaging, Aarhus University Hospital, Aarhus University, DK-8000 Aarhus, Denmark; 5 Danish Research Institute for Translational Neuroscience-DANDRITE, Nordic-EMBL Partnership of Molecular Medicine, Aarhus University, Department of Molecular Biology and Genetics and Department of Biomedicine, DK-8000 Aarhus C, Denmark; 6 Aarhus Institute of Advanced Studies (AIAS), Aarhus University, DK-8000 Aarhus C, Denmark

## Abstract

The Na^+^/K^+^-ATPases maintain Na^+^ and K^+^ electrochemical gradients across the plasma membrane, a prerequisite for electrical excitability and secondary transport in neurons. Autosomal dominant mutations in the human *ATP1A3* gene encoding the neuron-specific Na^+^/K^+^-ATPase α_3_ isoform cause different neurological diseases, including rapid-onset dystonia-parkinsonism (RDP) and alternating hemiplegia of childhood (AHC) with overlapping symptoms, including hemiplegia, dystonia, ataxia, hyperactivity, epileptic seizures, and cognitive deficits. Position D801 in the α_3_ isoform is a mutational hotspot, with the D801N, D801E and D801V mutations causing AHC and the D801Y mutation causing RDP or mild AHC. Despite intensive research, mechanisms underlying these disorders remain largely unknown. To study the genotype-to-phenotype relationship, a heterozygous knock-in mouse harboring the D801Y mutation (α_3_^+/D801Y^) was generated. The α_3_^+/D801Y^ mice displayed hyperactivity, increased sensitivity to chemically induced epileptic seizures and cognitive deficits. Interestingly, no change in the excitability of CA1 pyramidal neurons in the α_3_^+/D801Y^ mice was observed. The cognitive deficits were rescued by administration of the benzodiazepine, clonazepam, a GABA positive allosteric modulator. Our findings reveal the functional significance of the Na^+^/K^+^-ATPase α_3_ isoform in the control of spatial learning and memory and suggest a link to GABA transmission.

The *ATP1A3* gene encodes the Na^+^/K^+^-ATPase α_3_ subunit isoform. Mutations in the *ATP1A3* gene are associated with three related rare neurological disorders, rapid-onset dystonia-parkinsonism (RDP)^1^, alternating hemiplegia of childhood (AHC)^2^,^3^, and recently, cerebellar ataxia, areflexia, pes cavus, optic atrophy, and sensorineural hearing loss (CAPOS) syndrome^4^. The disorders arise from autosomal dominant mutations with variable penetrance^4^ and display overlapping symptoms that vary in severity, duration and frequency of occurrence^5^,^6^. In the case of AHC, affected patients typically present in the context of an acute onset of paroxysmal, episodic neurological symptoms that include hemiplegia, dystonia, ataxia, or seizures. Some symptoms may persist after resolution, such as neurodevelopmental delays, attention deficits, trunk instability, dystonia or ataxia^3^,^5^,^6^,^7^,^8^,^9^.

AHC patients are easily aroused and have frequent episodes of hyperactivity and mania. These episodes can be associated with high risk of injuries for the patients (Personal communications from Jeff Wuchich, and Dr. Hendrick Rosewich). Case reports suggest that between 18 and 53% of AHC patients develop epileptic seizures^7^,^8^. The level of cognitive deficits is often correlated with severity of epilepsy. Consequently, developmental delay and deficits in cognitive functions are very common among patients suffering from AHC^7^,^10^. Interestingly, the complexity of *ATP1A3*-related disorders is emphasized by the fact that clinically distinct neurological diseases seem to be caused by mutations in a single gene. In fact, amino acid substitutions in the same position have been shown to cause RDP or AHC. One example of this is the disease hot spot amino acid position 801, where currently four different mutations are known; D801Y causes RDP^1^,^11^ or AHC^12^, and D801N, D801E and D801V cause or AHC^2^,^3^,^13^,^14^,^15^.

In the central nervous system (CNS), the α_3_ isoform is expressed in neurons whereas the α_2_ subunit is expressed in glia, and the α_1_ subunit appears to be expressed ubiquitously^16^,^17^. Together, these Na^+^/K^+^-ATPase isoforms are responsible for maintaining the Na^+^ and K^+^ electrochemical gradients that determine cell resting membrane potentials, and support the electrical activity of excitable cells, as well as the transport of other ions and metabolites and driving neurotransmitter reuptake^18^. Although the role of the Na^+^/K^+^-ATPases in the etiology of neurological diseases is poorly understood, reduced Na^+^/K^+^-ATPase activity has been linked to conditions such as epileptic seizures and schizophrenia^19^,^20^. The distinguishing feature of α_3_Na^+^/K^+^-ATPases is their several-fold lower affinity for activation by cytoplasmic Na^+^ compared to that of α_1_Na^+^/K^+^-ATPases^21^. In rapidly firing neurons, therefore, when action potentials increase the intracellular Na^+^ concentration, [Na^+^]_i_, beyond levels saturating the “housekeeping” α_1_Na^+^/K^+^-ATPases, activation of α_3_Na^+^/K^+^-ATPases continues to increase as [Na^+^]_i_ rises. As [Na^+^]_i_ is linked to [Ca^2+^]_i_ through the Na^+^/Ca^2+^ exchanger, the α_3_ isoform thus protects neurons against catastrophic elevation of [Na^+^]_i_ and [Ca^2+^]_i_^22^ and general loss of the Na^+^ electrochemical gradient.

*Atp1a3* mouse models have provided insights into the role of the α_3_ isoform in neurological diseases. Currently, two knock-out and two knock-in mouse models have been reported. The heterozygous knock-out α_3_^+/KOI4^ mouse (*Atp1a3*^tm1Ling^) displayed spatial learning and memory deficits, hyperlocomotion and increased locomotor response to methamphetamine^23^. The α_3_^+/ΔE2-6^ mice knock-out mouse (*Atp1a3*^tm1.1Kwk^) showed increased sensitivity to kainate-induced dystonia and enhanced inhibitory neurotransmission of molecular-layer interneuron-Purkinje cell synapses in the cerebellar cortex^24^. The *Myshkin* mouse harbors the heterozygous I810N disease mutation in the *Atp1a3* gene (*Myshkin*, α_3_^+/I810N^, *Atp1a3*^Myk^), which in humans causes AHC^25^. The *Myshkin* mice are characterized by seizure activity^26^ and mania-like behavior, and showed increased response to amphetamine, similar to what has been reported for bipolar patients^27^ as well as motor dysfunction and cognitive impairments related to compromised thalamocortical functionality^28^. The D801N mutation is found in more than one third of AHC patients^3^. A recent study reported that heterozygous D801N knock-in mice (*Mashl*^+/−^, Mashlool, α_3_^+/I801N^)^29^ manifested several AHC-like symptoms including neuromuscular deficits, spontaneous recurrent seizures, and predispositions to kindling, to flurothyl-induced seizures and to Sudden Unexpected Death in Epilepsy (SUDEP)^29^.

To further address the complex genotype-phenotype relationship particular to the D801 amino acid position (the D801N/E/V mutations are associated with AHC whereas D801Y is associated with both RDP^1^,^11^ and AHC^12^) in the α_3_ isoform, the α_3_^+/D801Y^ mouse (*Atp1a3*^tm1Klh^) was generated and the general behavior and cognitive functions were explored. We found that α_3_^+/D801Y^ mice display *ATP1A3*-related symptoms such as hyperactivity, lower threshold for PTZ-induced epileptic seizures, and reduced hippocampus-dependent cognitive performance.

The hippocampus has been suggested to be the primary brain structure for spatial memory acquisition, memory storage and consolidation^30^. The hippocampal formation comprises dentate gyrus, the hippocampus proper and the adjacent parahippocampal cortices. The major excitatory input to the hippocampus arises from the entorhinal cortex via the perforant path that primarily terminates in the dentate gyrus. The dentate axons project to the CA3 region and from there the Schaffer collaterals convey the processed input to the CA1 area.

In spite of this, we did not observe any change in the excitability of CA1 pyramidal neurons in the α_3_^+/D801Y^ mice. The cognitive deficits were rescued by administration of the GABA positive allosteric modulator, the benzodiazepine clonazepam. The α_3_^+/D801Y^ model demonstrates a role in cognition comparable to the D801Y AHC manifestation, and will be suitable for investigations of disease mechanisms and development of therapeutic interventions.

## Results

### Non-Mendelian ratio and reduced α_3_ protein

**Figure 1:**
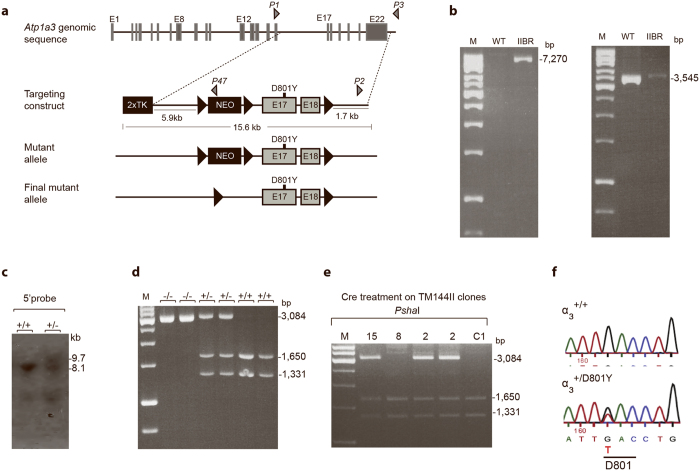
Generation and Targeting Strategy Screenings of the α_3_^+/D801Y^ knock-in mice. Diagram of the targeting strategy showing the oligonucleotide primers (grey triangles: P1, P2 and P3, P47, and P2) (**a**). PCR screenings verified homologous recombination in the IIBR clone, which was not observed for WT controls (**b**). Southern blotting analysis verified heterozygous homologous recombination in clone IIBR not observed for control (ES cells) (**c**). Partial Cre-excision of LoxP-NEO-LoxP cassette in the IIBR clone was verified by PCR and not observed for two controls (C1) (**d**) Confirmation of the third LoxP site was performed by a *Psha*I digest (a restriction site introduced with LoxP insertion) of a PCR product (using primers 5′-gtagccctgggattaaaggt and 5′gaagagaaggaggaatgaggg on genomic DNA). Treatment with the *Phsa*I enzyme gave in WT a non-digestable band of 2981 bp, and (+/−) gave 3084 bp, and 1650 bp and 1331 bp bands, whereas (−/−) gave 1650 and 1331 bp bands (**e**). Sequencing confirmed a heterozygous G → T base exchange in position 80 in the genomic DNA of a α_3_^+/D801Y^ mouse that was not present in WT (**f**). M; molecular DNA marker, bp; base pair, H_2_O; No template control where DNA was substituted with water.

**Figure 2:**
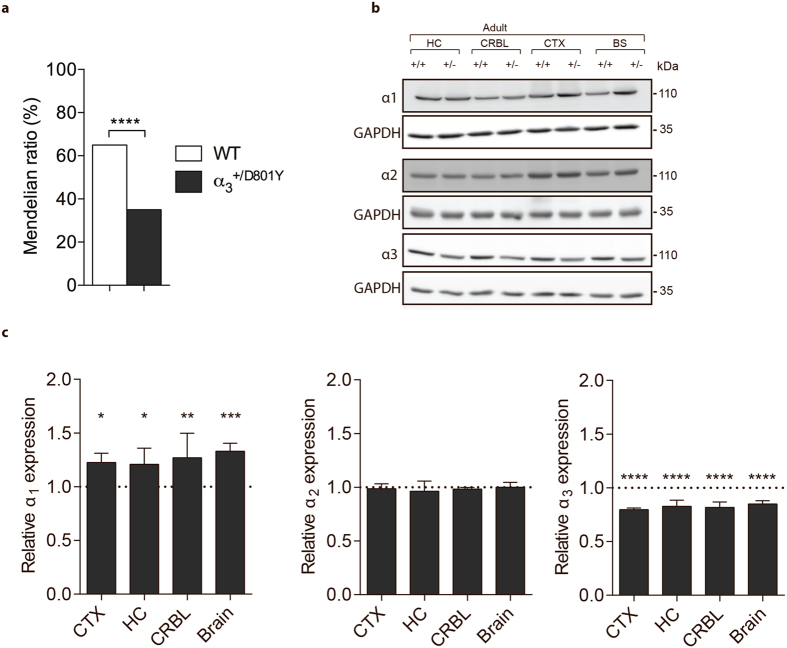
Basic characterization of the α_3_^+/D801Y^ mice. Boxplot showing skewed genotype ratio at weaning age (approximately postnatal day 21) (α_3_^+/D801Y^ mice N = 70, WT mice N = 130) (**a**). Representative Western blots illustrating α_1-3_ protein expression in α_3_^+/D801Y^ and WT mice in cortex (CTX), hippocampus (HC), cerebellum (CRBL) and in whole brain lysates (Brain) (full-length Western blots are shown in Supplementary Fig. 1) (**b**). Relative protein expression levels determined by densitometry showed a significant increase in □_1_ throughout the brain of α_3_^+/D801Y^ (N = 4). Expression of α_2_ was not affected (N = 3) whereas α_3_ expression was significantly reduced in α_3_^+/D801Y^ mice (N = 3) (**c**).

Upon generation of the α_3_^+/D801Y^ mouse model (Fig. 1), the line was back-crossed for >7 generations before testing. Analysis of the Mendelian distribution among 200 offspring, at 3 weeks of age, showed that 35% were genotyped as α_3_^+/D801Y^ (Fig. 2a), suggesting neonatal absorption and/or perinatal death. It should be noted that homozygous α_3_^D801Y/D801Y^ mice died at birth. The introduction of the D801Y mutation caused a 15% reduction in total α_3_ protein levels, but a 33% increase of the α_1_ protein levels, as tested by Western blotting (WB) analysis of whole brain, cortex, hippocampus, and cerebellum lysates from adult α_3_^+/D801Y^ mice relative to WT levels (Fig. 2b,c).

### α_3_^+/D801Y^ caused hyperactivity, but not anxiety

**Figure 3:**
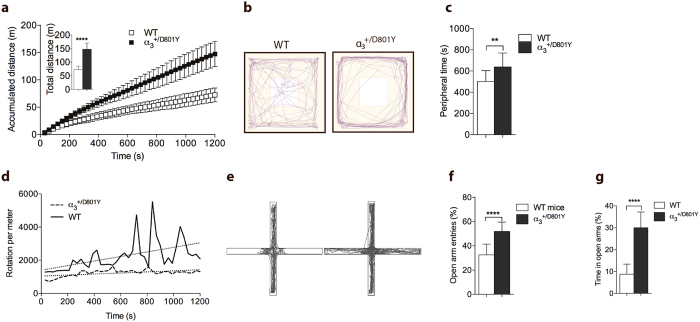
Increased spontaneous locomotor activity and altered exploration of the α_3_^+/D801Y^ mice. The α_3_^+/D801Y^ mice (N = 15) displayed hyperlocomotion (**a**) and traveled significantly longer in the open field test (a, insert) compared to WT mice (N = 14). Representative track plots of the first 3 minutes of open field exploration are shown in (**b**). The α_3_^+/D801Y^ mice remained significantly longer in the open field periphery than WT mice (**c**). After an initial habituation period, WT mice displayed increased meander defined as horizontal rotation per distance traveled. This behavior was completely absent in the α_3_^+/D801Y^ mice, here shown as the average score of 14 α_3_^+/D801Y^ and 14 WT mice (**d**). Representative track plots of elevated plus maze exploration are shown in (**g**). WT mice (N = 14) preferred to enter the closed arms whereas α_3_^+/D801Y^ mice (N = 15) showed no arm preference (**e**,**f**). The α_3_^+/D801Y^ mice occupied the open arms significantly longer than WT mice (**g**). All data shown are means ± SD *P < 0.05, **P < 0.01, ***P < 0.001, ****P < 0.0001.

In the open field test (OF), the α_3_^+/D801Y^ mice displayed hyperlocomotion relative to WT mice (Fig. 3a). After an initial habituation period of 8–10 minutes, WT mice showed a typical increase in horizontal rotation and meander. In contrast, α_3_^+/D801Y^ mice showed minimal habituation and almost exclusively changed direction when reaching the walls of the enclosure (Fig. 3b,d). Track plot analysis revealed a significant increase in time spent in the OF periphery (Fig. 3c). We hypothesized this to be a consequence of the low meandering rather than an indication of anxiety. In support, when tested in the elevated plus maze (EPM), the α_3_^+/D801Y^ mice did not discriminate between entering open and closed arms (Fig. 3e,f) and spent 240% more time in the EPM open arms compared to WT mice (Fig. 3g).

Thus, the α_3_^+/D801Y^ mice appears to reflect hyperactivtity and arousal to some degree, as they became highly agitated and displayed hyperactivity in response to handling and novel environments (described below in the Barnes Maze test), related to symptoms described for AHC patients.

### Reduced seizure threshold in the α_3_^+/D801Y^ mice

**Figure 4:**
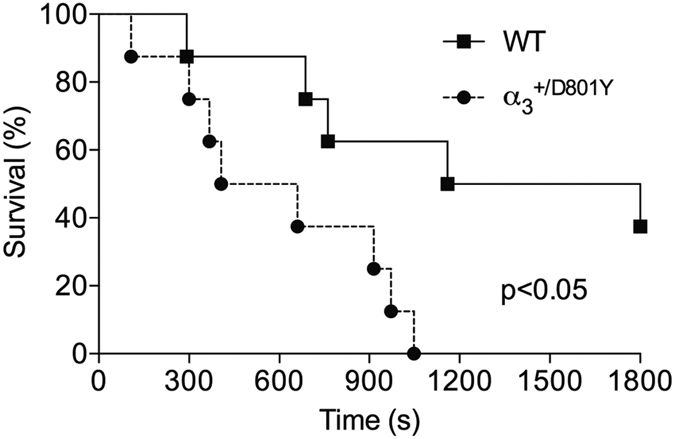
Reduced seizure threshold in the α_3_^+/D801Y^ mice. The α_3_^+/D801Y^ mice (N = 8) showed significantly reduced seizure threshold upon intraperitoneal injection with PTZ compared to WT littermates (N = 8) with no end point survival observed in the α_3_^+/D801Y^ mice.

Corresponding to the high rate of seizures reported for AHC patients, reduced Na^+^/K^+^-ATPase activity was shown to influence seizure activity in the *Myshkin* mouse model^26^ and contribute to SUDEP in the *Mashl*^+/−^ mouse model^29^. The α_3_^+/D801Y^ mice did not develop spontaneous seizures. To determine differences in subclinical seizure thresholds, α_3_^+/D801Y^ and WT mice were injected intraperitoneally with the non-competitive GABA antagonist, pentylenetetrazole (PTZ). PTZ induced a significantly stronger effect in the α_3_^+/D801Y^ mice as shown by the increased lethality in the α_3_^+/D801Y^ mice relative to WT mice (Fig. 4).

### The excitability of CA1 pyramidal neurons is not changed in the α_3_^+/D801Y^ mice

**Figure 5:**
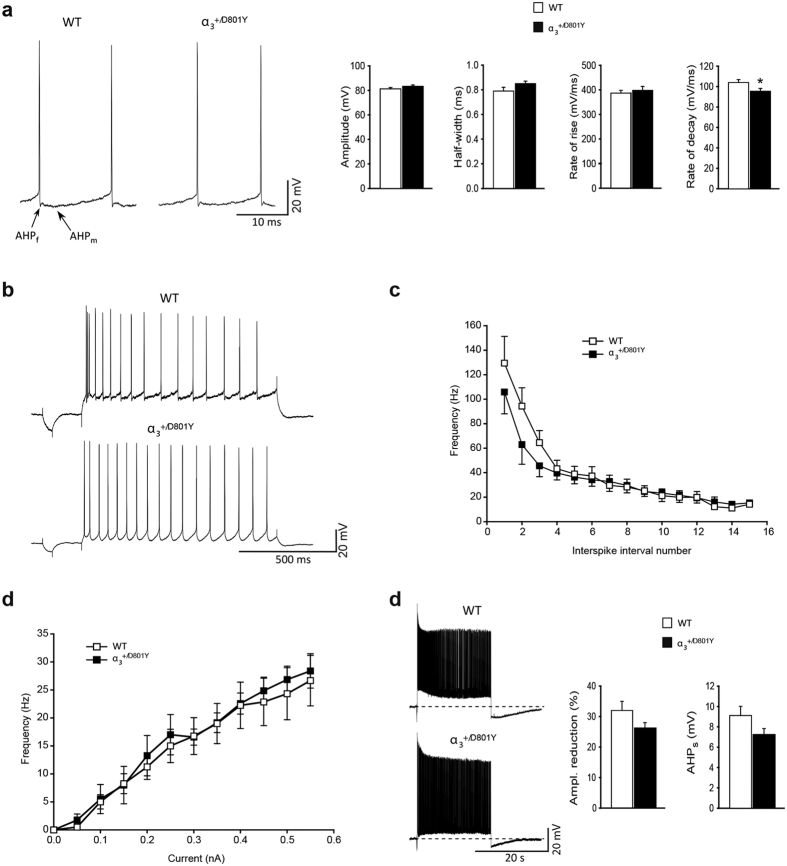
Electrophysiological characterization of hippocampal network. Electrophysiological properties of hippocampal CA1 pyramidal neurons from WT and α_3_^+/D801Y^ mice. Representative examples of action potentials (APs) evoked by a 200 ms depolarizing current pulse in WT and α_3_^+/D801Y^ mice (left) and their averaged quantitative parameters (right). The overall composition of the APs was similar and in both strains the APs were typically followed by a fast (f) and medium (m) afterhyperpolarization (AHP). There was no significant difference in the amplitude, half-width, or rate-of-rise between the WT and α_3_^+/D801Y^ mice; however, the rate of decay of APs was significantly slower in α_3_^+/D801Y^ mice (**a**). Representative examples of discharge behavior in response to a 1 s depolarizing pulses in WT (N = 13) and α_3_^+/D801Y^ (N = 11) mice. During repetitive firing, neurons from both strains displayed frequency accommodation (**b**). Plot of the averaged instantaneous discharge frequency *vs*. interspike interval number during repetitive firing of 16 Aps (**c**). Plot of discharge frequency *vs*. current intensity (**d**). Representative examples of responses to 20 s long suprathreshold current pulses in WT (N = 13) and α_3_^+/D801Y^ (N = 11) mice. In both strains there was relatively fast decline of the AP amplitude to a maintained level, and termination of the current pulse was followed by a pronounced slowly decaying afterhyperpolarization (AHP_s_). No significant difference was found in the average extent of AP amplitude decline (left) or in the magnitude of the AHP_s_ (right). The pre-pulse baseline potential was −67.4 ± 0.7 mV and −66.6 ± 1 mV in WT (N = 12) and α_3_^+/D801Y^ (N = 12) mice, respectively (**e**). All data shown are means ± SEM. *P < 0.05.

The hippocampal CA1 region is one of the brain areas in which PTZ induces highly synchronized epileptiform burst activity^31^. We therefore hypothesized that a reduced PTZ seizure threshold would be reflected in an increased excitability of CA1 pyramidal neurons. Using intracellular recordings in acute brain slices, no major difference in the basic membrane properties of CA1 pyramidal neurons in α_3_^+/D801Y^ and WT mice was found. The resting membrane potential (RMP) and input resistance (R_in_) were similar in α_3_^+/D801Y^ and WT mice (Supplementary Table 1). The threshold for induction of action potentials (APs) (Supplementary Table 1) as well as the overall composition of the APs were also similar (Fig. 5a) and typical of CA1 pyramidal neurons^32^. The only difference found with respect to the AP was a slight, but significant, reduction of the rate of decay (rate of repolarization) in α_3_^+/D801Y^ mice compared to WT (Fig. 5a). Depolarizing current pulses (1 s) induced trains of APs displaying frequency accommodation in WT and α_3_^+/D801Y^ mice (Fig. 5b). However, the amount of accommodation was less in α_3_^+/D801Y^ mice compared to WT mice, primarily due to a slower initial discharge rate in α_3_^+/D801Y^ mice compared to WT mice (Fig. 5c). No significant difference in steady-state electroresponsive behavior, measured as the frequency *vs*. current (*f*-I) relationship, was found between α_3_^+/D801Y^ and WT mice (Fig. 5d).

The α_3_ Na^+^/K^+^-ATPase has been suggested to serve as a “reserve” transporter activated when [Na^+^]_i_ is high, such as following prolonged high frequency discharge (reviewed in^22^). High frequency firing was evoked by a 20 s suprathreshold depolarizing current pulse. The assumption being that a reduced “reserve” capacity in α_3_^+/D801Y^ mice would lead to a higher rise in the [Na^+^]_i_ concentration, resulting in a more pronounced activity-dependent reduction of the amplitude of aPs. However, the activity-dependent reduction was similar between the two genotypes (Fig. 5e), as was the amplitude of the post-pulse slow afterhyperpolarization (Supplementary Table 1).

### Spatial learning and memory is reduced in a_3_^+/D801Y^ compared to WT littermates

More than 90% of AHC patients show signs of developmental delay or mental retardation^7^,^8^. Similarly, a recent care report showed that 90% of RDP patients with onset at or after 18 years had trouble learning in school^33^. The α_3_^+/D801Y^ mice were tested for spatial learning and memory performance using the Barnes Maze (Hippocampus-based spatial reference memory) and passive avoidance (amygdala- and hippocampus-based memory)^34^.

**Figure 6:**
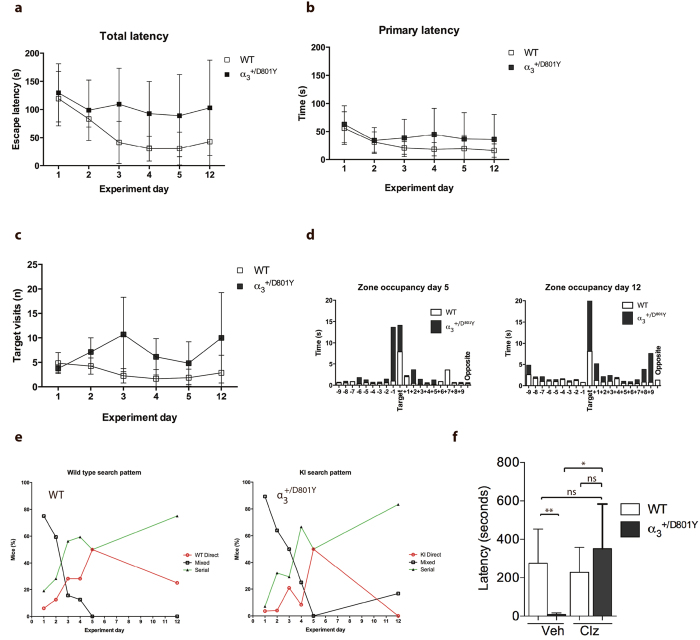
Reduced spatial memory and learning abilities in the α_3_^+/D801Y^ mice. In Barnes maze analysis, α_3_^+/D801Y^ (N = 6) and WT (N = 8) mice were monitored for (**a**) the time to enter the hidden tunnel i.e. total latency, (**b**) the time to first visiting the hidden tunnel i.e. the primary latency and (**c**) the number of target visits (**d**) Zone occupancy and (**e**) target hole strategies (Direct, serial and mixed). The passive avoidance test, (**f**) the α_3_^+/D801Y^ (N = 4) and WT (N = 6) mice differed in their response to clonazepam (Clz) compared to the vehicle treatment. All data shown are means ± SD. *P < 0.05.

In the Barnes Maze test, the mice were subjected to 4 training sessions per day for 4 days and subsequently a single test on day 5 and day 12. WT mice showed a significant reduction in latency to enter the escape tunnel (total latency). A similar learning curve was not observed for the α_3_^+/D801Y^ mice (Fig. 6a). Interestingly, the WT and α_3_^+/D801Y^ mice would reach the escape tunnel at the same time (primary latency) (Fig. 6b). However, once at the escape tunnel, the WT mice quickly entered, whereas the α_3_^+/D801Y^ mice would walk past multiple times before entering (Fig. 6c). Further analysis of zone occupancy confirmed that both genotypes after training spent the majority of time investigating the target area. Whereas WT mice specifically occupied the target zone on testing day 5 and 12, the α_3_^+/D801Y^ mice showed equal interest in the adjacent zone (−1) and remained outside for extended periods (Fig. 6d).

To assess the learning performance further, the strategy used to locate the target zone was analysed. Strategies were categorised as 1) Direct, where the mouse located the target tunnel or an adjacent hole using external cues 2) Serial, where the mouse seemingly chose a random hole and subsequently searched adjacent holes in a serial manner in a clockwise or counterclockwise direction or 3) Mixed, where the mouse displayed a more random search pattern and occasionally crosses the center of the circular platform.

During the first 4 days of training, it was evident that both genotypes shifted from employing the mixed search strategy to using either the serial or direct approach (Fig. 6e). At day 5, both genotypes showed a 50/50 utilisation of the serial and direct strategies. When testing the mice one week later on day 12, none of the α_3_^+/D801Y^ used the direct strategy and had reverted to using the serial or the mixed strategy. In contrast, the WT mice still utilised the direct and serial strategies (Fig. 6e).

### Fear memory is reduced in a_3_^+/D801Y^ compared to WT littermates

We used passive avoidance test to assess fear memory in the mice. Compared to WT mice, the α_3_^+/D801Y^ mice showed a significantly reduced latency to re-enter the dark compartment (Fig. 6f).

Alterations in inhibitory interneurons contribute to cognitive deficits associated with several psychiatric and neurological diseases. Inhibition by GABA receptors regulating neuronal activity helps to establish the appropriate network dynamics that support normal cognition^35^. To investigate if GABA transmission might be involved in the cognitive deficits observed in the mice, α_3_^+/D801Y^ and WT littermate mice were injected with the benzodiazepine, clonazepam and subsequently tested in the passive avoidance test. Clonazepam administrated intraperitoneally at 0.0625 mg/kg has previously been shown not to cause significant sedation or anxiolytic effect in the OF and EPM^36^. At this concentration, clonazepam completely normalized the performance of α_3_^+/D801Y^ mice in the passive avoidance test (Fig. 6f).

### Reduced number of hippocampal dentate gyrus granule cells in the α_3_^+/D801Y^ mice

**Figure 7:**
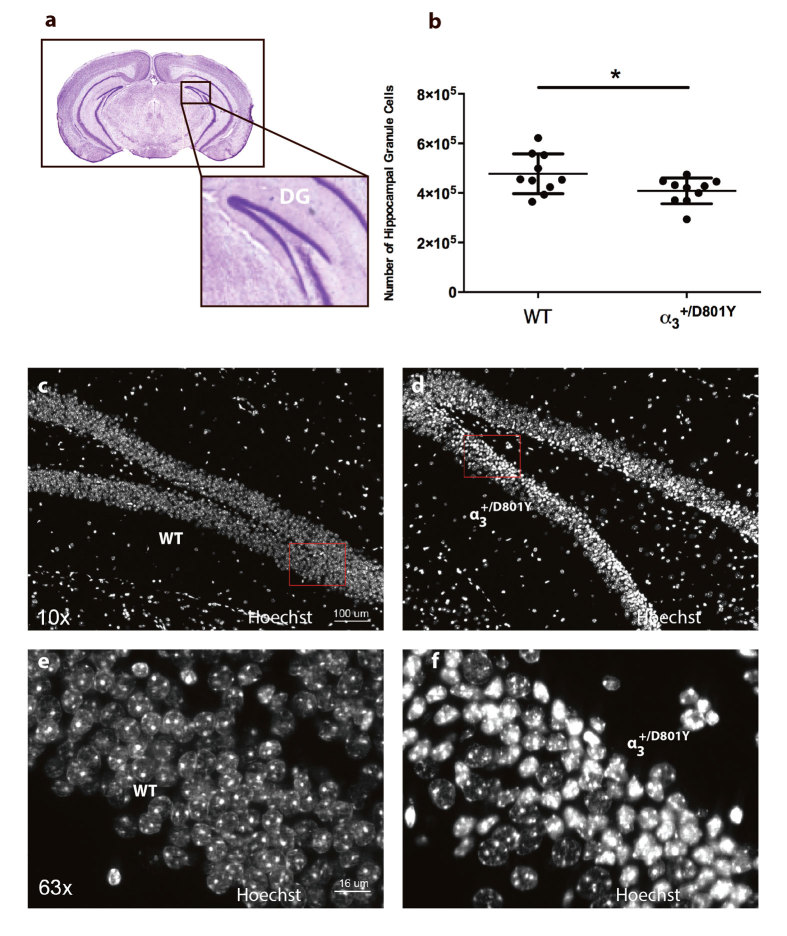
Number of neurons in hippocampus dental gyrus granual layer is reduced in the α_3_^+/D801Y^ mice. The number of neurons in the dentate gyrus granular layer (**a**) was significantly reduced in the α_3_^+/D801Y^ mice (N = 10, n = 408,217) compared to WT littermates (N = 10, n = 477,124) (**b**). All data shown are means ± SD. *P < 0.05. Hippocampal brain sections from the WT (**c**,**d**) and α_3_^+/D801Y^ (**e**,**f**) mice revealed a large number of pyknotic nuclei within the dentate gyrus granule cell layer in the α_3_^+/D801Y^ compared to WT littermates.

Since both memory tests pointed towards hippocampal defects, we performed histological examination of this brain region (Fig. 7a). Subsequent stereological counting showed a significantly reduced number of granule layer neurons in the dentate gyrus of the α_3_^+/D801Y^ mice (Fig. 7b).

Hippocampal brain sections from the α_3_^+/D801Y^ mice revealed a large number of pyknotic nuclei within the dentate gyrus granule cell layer compared to WT littermates (Fig. 7c–f), suggesting that the reduced number of granule layer neurons in the dentate gyrus of the α_3_^+/D801Y^ mice was partly due to this.

## Discussion

*ATP1A3* mutations have been recognized in infants and children presenting with diverse neurological symptoms^5^,^6^. In AHC patients, some of the most devastating symptoms include bouts of hyperactivity that may cause patients to injure themselves accidently and epileptic seizures that are associated with SUDEP and worsening of cognitive impairments. A recent case reports suggest that RDP patients also suffer from cognitive impairments, albeit to a lesser degree as many patients are able to attend and finish high school^33^.

The highly variable nature of *ATP1A3* diseases even for patients carrying the same mutation, has poised the theory that other factors such as genetic background, epigenetic modifications and environmental triggers influence the disease course.

Several studies show that α_1_ expression is influenced by changes in [Na^+^]^37^ and [K^+^]^38^, both are likely affected by reduced α_3_Na^+^/K^+^-ATPase activity. The compensatory upregulation of α_1_ protein in response to the reduced α_3_ protein expression in whole α_3_^+/D801Y^ brain lysates is therefore to be expected. Similar observations have previously been reported for the α_3_^+/KOI4^ mice^23^.

Homozygous α_3_^D801Y/D801Y^ mice died at birth, suggesting that α_1_ upregulation could not compensate for α_3_ loss at this stage, This is in accordance with suggestions that α_1_ and α_3_ differ not only in substrate affinity but also in localisation^39^ and that the CNS shifts from predominantly using the α_1_ and α_2_ isoforms during early development to α_1_ and α_3_ during post-natal development^26^.

In exploration-based tests for anxiety-like behavior, such as the OF and EPM, it can be difficult to dissociate symptoms of hyperactivity and attention deficits from anxiety-like behavior, as they may interfere with spontaneous exploratory locomotion. Although the α_3_^+/D801Y^ mice spent significantly more time in the OF periphery, we propose this to be a direct consequence of the lack of meander rather than a trait of anxiety. In support, the α_3_^+/D801Y^ did not discriminate between entering open and closed arms in the EPM and spent significantly more time than WT mice exploring the EPM open arms. In further support of this hypothesis, similar behavior was described for an attention deficit mouse model^40^,^41^.

The hyperactive phenotype observed in the OF was induced response to handling and novelty in general. This was particularly evident during the memory tests, where repeated handling and the stressful environment caused some α_3_^+/D801Y^ mice to become so agitated that they would jump off the testing platforms repeatedly and hurt themselves. These mice were omitted from the study. We believe this behavior could reflect in some degree the hyperactive and manic episodes of AHC patients.

Reduced Na^+^/K^+^-ATPase activity has previously been described in human epilepsy patients^20^. There is a growing appreciation that genetic factors contribute to the etiology of seizures^42^. With the recent case report of D801Y patient diagnosed with late onset mild AHC, it is possible that other symptoms of *ATP1A3*-related disorders are further affected by the genetic background.

The *Myshkin* and Mashl^+/−^ mice were originally maintained in the 129S1/SvImJ and 129 SV background, respectively, and developed spontaneous tonic-clonic seizures and epileptic discharges as well as a SUDEP-like phenotype^26^,^29^.

Despite a reduced PTZ seizure threshold, we did not observe spontaneous seizures in the α_3_^+/D801Y^ mice. Spontaneous seizures have also not been reported for the α_3_^+/KOI4^ and α_3_^+/ΔE2-6^ mice^23^,^24^. Supporting a strong contribution from genetic background, the latter two *Atp1a3* mouse models were maintained in the seizure resistant C57BL6/J strain^43^. Given the close relationship to the C57BL/6JRJ strain, it is likely that some of the same genetic modifiers play a role in the seizure phenotype of the α_3_^+/D801Y^ mice. In further support of this theory, the *Myshkin* mice became resistant to stress-induced seizures once maintained in the seizure-resistant C57BL/6NCr strain for 20 generations^27^.

Electrophysiological measurements in acute brain slices from naïve animals showed only minor differences between the genotypes. This cannot account for the decreased seizure threshold. It therefore seems unlikely that the increased excitability of the α_3_^+/D801Y^ mice can be explained by changes in the basic membrane properties of the CA1 pyramidal neurons.

The hippocampus regulates the generation of long term memory^44^ and spatial learning^45^ and has been shown to be critical for spatial memory in human subjects with hippocampal damage (reviewed in^46^). Together, the presented memory tests suggest an essential role of hippocampal α_3_Na+/K+-ATPase in consolidating particularly long-term spatial memory and fear-dependent memory, whereas short-term memory seemed less affected.

The Barnes maze test showed that the α_3_^+/D801Y^ mice took significantly longer time to enter the target tunnel. This difference was not caused by an inability of the α_3_^+/D801Y^ mice to locate the tunnel, but rather the fact that the α_3_^+/D801Y^ mice spent upwards of four times longer at the entrance before entering. The Barnes maze test relies on the instinct of mice to escape a brightly lit area and to seek protection in a tunnel. The mouse is guided towards the tunnel via external cues mounted on the surrounding walls. The lack of discrimination between open and closed arms in the EPM would suggest that the instinct to seek cover is suppressed in the α_3_^+/D801Y^ mice. Similar behavior has previously been described in the dopamine transporter knockout mouse, a mouse model for attention-deficit/hyperactivity disorder (ADHD) and schizophrenia-like behavior^40^,^41^.

We propose that the increased total latency is a consequence of failure to process the stressful surroundings of the Barnes maze, originally created to drive the mice into the tunnel.

Barnes maze track plot analysis revealed that the mice irrespective of genotype employed similar search strategies during the first 4 days of training: At day 5, both genotypes used the serial and direct strategies to an equal extent, suggesting that the α_3_^+/D801Y^ mice have functional short-term spatial memory. The direct approach requires that the mice navigate using external cues, confirming that the vision of the α_3_^+/D801Y^ mice was intact.

At day 12, the α_3_^+/D801Y^ mice no longer used the direct strategy and reverted to predominantly using the serial and to a lesser extent the mixed approach. This suggests that their long-term spatial memory is affected. Primary latencies were not affected by this change in tactics. We initially expected a compensatory effect of the hyperlocomotion, previously observed in OF, but average speed for both genotypes was similar (not shown).

The α_3_^+/D801Y^ showed reduced fear memory in the passive avoidance test. It was clear that given the right environment, the mice were fully capable of entering a dark compartment. A likely explanation for poor performance of the α_3_^+/D801Y^ in the Barnes maze is therefore that the stressful environment interfered with the decision-making of the α_3_^+/D801Y^ mice.

The reduced spatial learning and memory abilities in the α_3_^+/D801Y^ mice suggested dysfunction of the amygdala and hippocampal brain regions. Histological examination revealed a large number of pyknotic nuclei within the granule layer of the dentate gyrus. Similar nuclear morphology has previously been described in ouabain-treated cultured cortical neurons undergoing hybrid cell death^47^. Furthermore, similar hippocampal pathology has previously been reported in rats injected with ouabain into the hippocampus^48^ and in a rat model of pilocarpine-induced chronic epilepsy^49^, thus strengthening the link between Na^+^/K^+^-ATPases perturbations and seizure.

The α_3_ isoform is highly expressed in the GABAergic basket cells in the subgranular zone^17^ that are responsible for proliferation of granule cells during early development. It is therefore likely that the reduced number of granule cells in the dentate gyrus is the result of skewed apoptosis/proliferation in this region and that this is directly affected by the reduced α_3_ activity.

Memory deficits were noted in the heterozygous knock-in mouse models *Myshkin*^50^ and Mashl^+/−^29 as well as the heterozygous knock-out model α_3_^+/KOI4^ mice^23^, strongly supporting the role of the α_3_ in hippocampus-dependent cognition. The α_3_^+/KOI4^ mice showed reduced expression of the N-methyl-D-aspartic acid receptor (NMDR)^51^. The NMDR has a well-documented role in the formation of several memories, including spatial, olfactory and contextual memory^52^. NMDA receptor expression was reduced by applying ouabain to cerebellar neurons^53^. Reduced NMDA receptor NR1 expression was described in homozygous E18 *Myshkin* mice, but not in heterozygous E18 and adult *Myshkin* mice^26^.

The co-expression of the α_3_ isoform in GABAergic neurons suggests an association between the Na^+^/K^+^-ATPase and GABA transmission. The role of GABA_A_ receptors in learning and memory and neurological disorders is well documented (recently reviewed^54^). In particular, GABA regulates oscillations implicated in learning and memory, by generating synchronized inhibitory postsynaptic potentials. Dysfunctions caused by the α_3_ isoform has previously been linked to GABA transmissions in the *Myshkin* mouse^26^ and aberrant cerebellar function in the α_3_^+/ΔE2-6^ mice^24^. To test if increasing GABAergic transmission could improve learning, the α_3_^+/D801Y^ mice was treated with the benzodiazepine, clonazepam. A single injection rescued passive avoidance performance and thus fear-dependent memory. Interestingly, similar effects were recently reported for the *Scn1a*^+/−^ mouse model for Dravet’s syndrome^36^, a disease where GABAergic neurotransmission is specifically impaired by a mutation in the SCN1A gene encoding voltage-gated sodium channel Na_V_1.1. Interestingly, the *Scn1a*^+/−^ mice also exhibited hyperactivity, and impaired context-dependent spatial memory. Supporting the hypothesis that GABA is indeed a major contributor towards *ATP1A3*-related diseases is the fact that GABA_A_ receptors are implicated in childhood epilepsy^55^, and patients with temporal lobe epilepsy exhibit altered expression of the mRNA encoding the GABA_A_ receptor in several hippocampal sub regions^56^,^57^,^58^. It was clear that PTZ, as a temporal lobe epileptic inducer, lowered the seizure threshold in the α_3_^+/D801Y^ mice compared to WT mice, supporting the role of GABA.

The effects of clonazepam are associated with allosteric activation of the ligand-gated GABA_A_ receptor^59^. The current note of GABA_A_ receptor complex subunits is that the GABA_A_ α5 subunit might be implicated in memory and learning, however, the GABA_A_ subtype alteration in the α_3_^+/D801Y^ mice remains to be elucidated.

The results presented here strengthen the ongoing debate of the complexity of the *ATP1A3*-related diseases: Why some mutations are specific to RDP, AHC or CAPOS, and why the same mutations may produce intermediate symptoms could give rise to so very different disease courses. Given that the D801Y mutation has been shown to cause RDP and AHC in human patients, the α_3_^+/D801Y^ mice may present as a unique platform to investigate this further. *ATP1A3*-related diseases have no effective treatments^5^. It is therefore interesting that the cognitive deficits in the α_3_^+/D801Y^ mice could be reverted by a single low dose of clonazepam. This novel mouse model could be helpful for future developments of targeted treatments in neuropharmacology and memory functions^60^.

## Methods

### Generation of the α_3_^+/D801Y^ KI mouse model

#### Vector preparation

Preparation of LoxP-NEO-LoxP (PCR sing LPN_Bcl_F: 5′-cggtgatca-ataacttcgtatagca and LPN_Bcl_R: 5′-cggtgatca-gcctgctattgtcttc) and 2xTK cassette were performed as described^61^. The 2xTK cassette (3652 bp) was cloned into Strataclone^TM^ blunt PCR cloning vector, pSC-B (3.5 kb) using the *Sac*I restriction enzyme site (generating the pSC-B-2xTK). The 5′ end 5921 bp PCR product (A3 I18 sac 2R: 5′-GCAACCAAAGTCGAGACTCC and I16 F not: 5′-GAAGGCCTCCTCTCCTGACAT) was cloned into the pSC-B-2xTK (generating the pSC-B-2xTK-5HOM), and a LoxP site- containing PCR product (LP_BstX1_F: 5′-cggccannnnnntgg-ataacttcgtatagca and LP_BstX1_R:5′- cggccannnnnntgg-ataacttcgtataatgt) was inserted using a *Bst*X1 restriction enzyme (pSC-B-2xTK-5HOM-LP). The LoxP-NEO-LoxP insert was cloned into the pSC-B-2xTK-LP vector using the *Bcl*1 restrition enzyme (generating the pSC-B-2xTK-5HOM-LP-LPNLP). The 1660bp 3′ end PCR product (I21 R sac2: 5′-GCCCAGGCTAACCTCAAACT and A3 I 18 sca2 F: 5′-GCAACCAAAGTCGAGACTCC) was cloned into Strataclone^TM^ blunt PCR cloning vector, pSC-B (3.5 kb) (generating the p-SC-B-3HOM construct).

Site-directed mutagenesis was performed to introduce the D801Y mutation using the following primers to introduce this mutation, atp1a3-blp-mut-F 5′-cgcttagcagcacatccccctttc and atp1a3-blp1-mut-R 5′-tgctaagcgattaattgggtaac. The pSC-B-2xTK-5HOM-LP-LPNLP and p-SC-B-3HOM was combined using a *Cla*I site, generating the *Atp1a3* D801Y gene targeting vector (15.642 bp).

CJ7 ES cells (from 129 S1/Sv)^62^ were electroporated with the linear targeting vector and double selected with G418 (350 μg active substance/ml) and FIAU (0.5 μM) (Fig. 1a). Targeting efficiency was 3/360 double resistant clones Homolog recombination in ES cell clone TM144 II B12 was confirmed by 5′end and 3′end PCR’s using primers located to the NEO cassette sequence (P47) and Atp1a3 sequence specific primers (A3_F_5_3, A3.3.R and A3.1_F4) located to *Atp1a3* sequences not included in the gene targeting vector, as follows; **5**′**arm**: 47 (NEO): 5′-caggactgtacaaatggaag and A3_F_5_3:5′-gcttccgtgctcctgtgt- Product size 7270 (Fig. 1b). The **3**′**arm:** A3.3.R 5′-taatcgaggtgtgggagagg **and** A3.1_F4 5′-aagaaatccatcgcctacac. Product size 3545 (Fig. 1a). Homolog recombination in ES cell clone TM144 II B12 was confirmed by Southern blotting with a 5′ probe binding annealing to the *Atp1a3* sequence in the 5′end of the targeting construct (Supplementary figure 1C). 5–10 μg genomic DNA was digested with *Bst*X1, and probed with a 5′probe prepared using the following primers; A3_5_F gagaccggttccatctcca A3_5_R gatccaggcctaagcttcct (Probe product size 1010 bp) (Fig. 1c).

Confirmation of LoxP3 site was performed by PCR using the following primers; A3 1 1 C1: 5′-GTAGCCCTGGGATTAAAGGT and A3Rev-RC: 5′gaagagaaggaggaatgaggg. PCR of (+/+) allel = 2981 bp and PCR of (+/−) allel = 3084 bp. Digestion with restriction enzyme *Psha*I, a site that was deleted introducing the third LoxP site, allowed distinction, (+/+) gave 1650 and 1331 bp, and (+/−) gave 3084 bp, and 1650 bp and 1331 bp, whereas −/− gave 3084 bp only (Fig. 1d). *PshA*1 digest of 3′arm 3545 bp PCR product: Correct recombination; 3545, 3096, 449 and WT, 3096, 449 bp (Data not shown).

#### Partial cre of transfected ES cells

The NEO cassette was removed by partial Cre-enzyme treatment leaving a single LoxP site in intron 16 obtained by transfecting TM144 II B12 ES cells with linearized Cre-enzyme encoding plasmid, as described^61^. Successful partial Cre-enzyme treatment was confirmed for the TM144 II B12 cre 2 and 15 clone by PCR (A3.1.1.C1 and A3.Rev.RC) (data not shown) and *Psh*I digestions of the PCR product generated specific band patterns (Fig. 1e) (No cre: 1650 bp and 1331 bp, full cre; 1902 bp, 1650 bp and 1331 bp, partial cre; 3084 bp, 1650 bp and 1331 bp). The TM144 II B12 cre 8 revealed at non-cre event. The final introduction of the D801/Y mutation was confirmed by DNA sequencing (Fig. 1f). Transgenic α_3_^+/D801Y^ knock-in (KI) mice (*Atp1a3*^tm1Klh^) were crossed to c57/BL6JRj (Janvier) background, and heterozygous α_3_^+/D801Y^ mice were identified by qPCR genotyping (described below).

#### Genotyping

Heterozygous α_3_^+/D801Y^ mice were genotyped by High Resolution Melt analysis (Roche, Basel, Switzerland on a lightcycler 96 (Roche, Basel, Switzerland). using forward primer tcatggctaacatcccactg and reverse primer agtagcagccaggcttacca (Sigma-Aldrich; Schnelldorf, Germany).

### Animal ethics and conditions

All *in vivo* studies were performed using α_3_^+/D801Y^ mice and WT obtained by crossing α_3_^+/D801Y^ mice (generation N ≥ 8) with C57BL/6JRj mice (Janvier). Mice were kept at a daily 12 h light/dark cycle. Tests were performed during the light cycle. The mice were maintained as heterozygotes (α_3_^+/D801Y^) since homozygotes were neonatally lethal. Mice used for all experimental procedures are between 10–20 weeks of age. Experimental protocols involving mice, performed at Aarhus University were performed according to the Danish national and Institutional regulations and approved by the Animal Experiments Inspectorate under the Danish Ministry of Justice (permit numbers 2012-15-2934-00621, 2013-15-2934-00815 and 2014-15-2934-01029) to KLH.

### Basic Characterization

#### Western blot

The protocol for Western blot was performed as previously described^61^. Primary antibodies (anti α1 1:2000 (a6f-c, DevelopmenalStudies Hybridoma Bank), anti α2 1:1000 (07674, EMD Millipore, US), anti α3 1:1000 (06172, EMD Millipore, US), anti DAT 1:500 (MAB369, EMD Millipore, US), anti TH 1:2000 (AB152, EMD Millipore, US) and GAPDH 1:1000 (ab9485, Abcam, Cambridge, UK)) were incubated overnight at 4 °C. Next day, membranes were incubated with horseradish peroxidase-conjugated secondary antibodies (swine anti-rabbit 1:2000 (Dako, Glostrup, Denmark), rabbit anti-mouse 1:2000 (Dako, Glostrup, Denmark)) for 1 hour at room temperature. Visualization was done with a LAS 3000 imager (Fujifilm, Tokyo, Japan) using Amersham ECL Western Blotting Detection Kit (GE Healthcare, Buckinghamshire, UK) as the detection reagent. ImageJ version 1.48 v was used for densitometric analysis of the Western blots.

### *In vitro* electrophysiology

#### Preparation of brain slices

Male mice were anesthetized with isoflurane and decapitated. The brain was removed and quickly placed in dissection medium (in mM; 120 NaCl, 2 KCl, 1.25 KH_2_PO_4_, 6.6 HEPES acid, 2.6 NaHEPES, 20 NaHCO_3_, 2 CaCl_2_, 2 MgSO_4_ and 10 D-glucose, bubbled with 95% O_2_ and 5% CO_2_) at 4 °C. The hippocampus was dissected free, and 400 μm slices were cut using a McIlwain tissue chopper. One slice was immediately transferred to the recording chamber, where it was placed on a nylon-mesh grid at the interface between warm (31–32 °C) aCSF (in mM; 124 NaCl, 3.25 KCl, 1.25 NaH_2_PO_4_, 20 NaHCO_3_, 2 CaCl_2_, 2 MgSO_4_ and 10 D-glucose, bubbled with 95% O_2_ and 5% CO_2_, pH 7.3) and warm humidified gas (95% O_2_, 5% CO_2_). Perfusion flow rate was 1 ml/min. The slice rested for at least one hour before electrophysiological recordings were started. The remaining slices were stored in dissection medium bubbled with 95% O_2_ and 5% CO_2_ at room temperature.

#### Electrophysiological recordings

Intracellular recordings were obtained using borosilicate glass electrodes (1.2 mm OD; Clark Electromedical, Pangbourne, UK) filled with 4 M K^+^ acetate and placed in stratum pyramidale in area CA1. Conventional recording techniques were employed, using a high-input impedance amplifier (Axoclamp 2A, Molecular Devices, USA) with bridge balance and current injection facilities. Signals were digitized online using a Digidata 1440 interface and transferred to a computer for analysis employing pCLAMP (version 10, Molecular Devices). Inclusion criteria were a stable resting membrane potential (RMP) ≤ −50 mV, a membrane input resistance (R_in_) ≥ 10 MΩ and an action potential amplitude ≥70 mV.

Once an intracellular recording was established, a series of stimulation protocols were employed both at RMP and after the membrane potential was clamped at −65 and −70 mV.

#### Analysis

R_in_ was evaluated from the current-voltage relationship close to RMP; the AP threshold was measured using short (4 ms) depolarizing current pulses of increasing intensity; the rates of rise and decay of the AP were taken as the maximal slopes; the frequency *vs*. current (*f*-I) relationship was estimated with 500 ms depolarizing current pulses of increasing intensity. Frequency accommodation was estimated as the variance of interspike duration during repetitive firing of 16–19 APs evoked by a 1 s depolarizing current pulse from a baseline potential of −65 mV. The time-dependent decay in AP amplitude during 20 s repetitive firing was estimated using the following formula: (1^st^ AP – last AP)/1^st^ AP.

Unless otherwise indicated, values are given as mean ± S.E.M, and the unpaired Student’s t-test or Mann-Whitney rank sum test were used for statistical evaluation. For multiple comparisons the two-way ANOVA was used. The level of significance was set at 5%.

### Behavioral paradigms

Experiments were conducted blinded using α_3_^+/D801Y^ mice and age-matched WT littermates. Mice were transferred to the test room one hour prior to testing for acclimation and tests were performed 1–2 days after last cage change. Behavioral apparatuses were cleaned between tests in 70% EtOH and only one gender was tested per experiment.

#### Open field

Mice were placed in a 50 × 50 cm open field (Stoelting Europe; Dublin, Ireland) and monitored for 20 minutes using the ANY-maze software V4.99 (Stoelting, USA). Three zones were defined: A peripheral zone measuring approximately 8 cm from the walls, an intermediate zone extending another 8 cm into the apparatus and a center zone.

#### Elevated plus maze

Entries into the open and closed arms of the elevated-plus maze (Stoelting Europe; Dublin, Ireland), time spent in these arms, as well as distance traveled was recorded for 10 minutes using the ANY-maze software (Stoelting Company).

#### Barnes Maze and passive avoidance were performed as recently described

Trials were recorded by using computerised^34^ tracking/image analyser system and analysed using the ANY-maze tracking system (Stoelting Company). The following parameters were recorded: errors, distance from tunnel, search strategy and time that the mouse took to escape into the tunnel i.e. total latency. Errors were defined as nose pokes and head deflections over any hole that did not have the tunnel. The search strategies were determined by examining each mouse’s daily session and defined in to three categories: (1) Direct (spatial): Moving directly to target hole or to an adjacent hole before visiting the target. (2) Mixed: Hole searches separated by crossing through the center of the maze or unorganised search. (3) Serial: The first visit to the target hole was preceded by visit at least two adjacent holes in serial manner, clockwise or counter clockwise direction^63^.

The passive avoidance test was initiated on the acquisition day (A). The mouse was placed in a brightly lit compartment with an electronically controlled door leading into a dark compartment. The latency (s) was recorded for the mouse to enter the dark compartment. Once in the dark compartment, the door closed and the mouse received an electric shock (0.42 mA for 1 s). Twenty-four hours later (retention day, R), the mouse was reintroduced to the same brightly lit compartment and the latency to enter the dark compartment was recorded as an indicator of memory of the shock.

#### Clonazepam passive avoidance rescue

Thirty minutes prior to passive avoidance training, the mice received 0.0625 mg/kg clonazepam intraperitoneally (Roche, Hvidovre, Denmark) dissolved in 0.9% sterile saline (vehicle) or vehicle alone.

#### PTZ seizure threshold

Mice were given 75 mg/kg pentylenetetrazole (Sigma-Aldrich; Schnelldorf, Germany) or 0.9% NaCl vehicle IP and monitored and video-recorded for 30 minutes after which they were euthanized.

### Brain sampling and immunohistochemistry

Mice were deeply anesthetized with an overdose of pentobarbital. Approximately 0.05 mL per 10 g body weight pentobarbital was given intraperitonally (50 mg/mL pentobarbital, Aarhus University Hospital). When sedated, the mice were fixed upon a polystyrene board, and their chests were cut open with a blunt pair of scissors. The mice were perfused with 20 mL ice cold phosphate buffered solution (PBS) transcardially and subsequently with 20 mL ice cold 4% paraformaldehyde (PFA) in PBS. The brains were carefully dissected out and post-fixed in 4% PFA, PBS 4 °C over night (ON). The brains were cut in half following the midline and the olfactory bulb and cerebellum were dissected from the right hemispheres, and these halves were used for sampling. The left hemispheres were saved for later studies.

The tissues were infiltrated in paraffin using a Shandon Citadel^TM^ Tissue Processor (Thermo Scientific).

The right hemispheres were coronally sliced on a microtome at a thickness of 30 μm. A stereotactic atlas of the mouse brain (Paxinos and Franklin, 2003, second edition) was used to identify a region juxtaposed to the hippocampus in order to have a visual guideline for initiating the collection of the sections. The point of reference chosen was the dorsal third ventricle at approximately Bregma −0.22 mm that was approximately situated 0.70 mm frontally to the hippocampus. The hippocampus stretches from Bregma −0.94 mm to −3.88 mm according to the atlas. Every second section was collected on Superfrost ^®^ Plus Microscope Slides (Thermo Scientific) spanning 4 series. One of the series was used for stereological analysis. Microscopic examination ensured that the hippocampus had been fully sectioned. After collection of the sections, the slides were put in the oven at 65 °C for 30 min. Sections were then deparaffinised Xylene (2 × 15 min), 99% EtOH (3 × 5 min), 96% EtOH (3 × 5 min) and 70% EtOH (2 × 5 min). Sections for stereological analysis were stained with toluidine blue, subsequently dehydrated and cover slipped. Sections for histological assessment were stained with Hoechst and cover slipped.

### Stereological analysis

The optical fractionator was used as counting methodology, and quantitative stereological analysis was performed by the same person who was blinded to the phenotype of the mice.

#### Equipment

Counting was done on a computerized optical microscope (Olympus BX50) equipped with a motorized stage and focus control system (Prior Scientific, ProScan™ III). A highly specialized software program (Visiopharm integrator system version 4.5.1.324) was used for counting.

#### Sampling

Every second section was collected spanning 4 series in order to have at least six sections of the hippocampus in every series. Counting was only done on one of them giving a section sampling fraction, *ssf*, of 1/8. The final tissue thickness after shrinkage was determined to be approximately 25 μm, which allowed the height of the disector to be 15 μm with safeguard zones of 5 μm at the top and 5 μm at the bottom. The top of the tissue is excluded from the counting as sectioning can extract parts of cells close the sectioning plane^64^. The height of the disector is used to calculate the height sampling fraction, 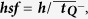 where 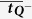 is the Q^−^-weighted mean section thickness which can be calculated as:


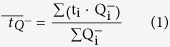


*t*_*i*_ is the local section thickness placed in the *i*^th^ counting frame with a disector count of 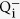
65.

An unbiased counting frame with an area, a = 76 μm^2^, was superimposed on each field of view within the area of the granular layer of dentate gyrus. The step length was 100 μm in both the x and y plane and hence the sampling area fraction was:


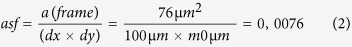


In order to achieve a sample estimate, N, with a CE less than 0.1, the number of sections, the step-length and the area of the counting frame were dimensioned so that ~200 cells were counted per series.

#### Counting

The GrDG of each hippocampus present on a slide was delineated using a 4× objective at a final magnification of 135×. Subsequently, meander counting was performed using the 100× objective at a final magnification of 3366×. At each field of view, the microscope was slowly focused down from top to bottom of the section. All non-pyknotic neurons sampled by the 2D unbiased counting frame and located within the height of the optical disector were counted. Pyknotic cells were counted as a separate population of cells.

### Statistical analysis

All data were shown as mean ± s.d. (or SEM) and statistical analyses were done using Graphpad Prism version 5.01 or 6.03 (GraphPad Software Inc, La Jolla, CA, USA or the R software (R Foundation for Statistical Computing, Vienna, Austria)^66^ (Supplementary Table 1). The obtained male and female mice data were only pooled when this was statistically validated (*P* < 0.05), and all statistical tests and the *P*-values obtained are presented in Supplementary Table 1.

## Data availability

The α_3_^+/D801Y^ mouse model is available through a Material Transfer Agreement (MTA).

## Additional Information

**How to cite this article**: Holm, T. H. *et al*. Cognitive deficits caused by a disease-mutation in the α_3_ Na^+^/K^+^-ATPase isoform. *Sci. Rep*. **6**, 31972; doi: 10.1038/srep31972 (2016).

## Supplementary Material

Supplementary Information
