# Supplementary material for: Cognitive deficits caused by a disease-mutation in the α3 Na+/K+-ATPase isoform
Source: Sci Rep. 2016 Aug 23;6:31972. doi: 10.1038/srep31972 (PMC4994072; doi:10.1038/srep31972)
Supplement: Supplementary Information [file srep31972-s1.pdf]

Cognitive deficits caused by a disease-mutation in the α_3 Na^+/K^+-ATPase isoform

Thomas Hellesøe Holm[1,2], Toke Jost Isaksen[1,2], Simon Glerup[1], Anders Heuck[1,2], Pernille Bøttger[1], Ernst-Martin Fuchtbauer[3], Steen Nedergaard[1], Jens Randel Nyengaard[4], Mogens Andreasen[1], Poul Nissen[2,3,5], and Karin Lykke-Hartmann[1,2,6,*]

Affiliations:
[1]Aarhus University, Department of Biomedicine, DK-8000 Aarhus, Denmark.
[2]Centre for Membrane Pumps in Cells and Disease-PUMPKIN, Danish National Research Foundation, Aarhus University, Department of Molecular Biology and Genetics, DK-8000 Aarhus C, Denmark.
[3]Aarhus University, Department of Molecular Biology and Genetics, DK-8000 Aarhus, Denmark.
[4]Stereology and Electron Microscopy Laboratory, Center for Stochastic Geometry and Advanced Bioimaging, Aarhus University Hospital, Aarhus University, DK-8000 Aarhus, Denmark
[5]Danish Research Institute for Translational Neuroscience-DANDRITE, Nordic-EMBL Partnership of Molecular Medicine, Aarhus University, Department of Molecular Biology and Genetics and Department of Biomedicine, DK-8000 Aarhus C, Denmark.
[6]Aarhus Institute of Advanced Studies (AIAS), Aarhus University, DK-8000 Aarhus C, Denmark.

* Correspondence: Dr K Lykke-Hartmann, Aarhus Institute of Advanced Studies and Department of Biomedicine, Aarhus University, Wilhelm Meyers Allé 4, DK-8000 Aarhus C, Denmark, Tel: +45-29390558, Fax: +45-86128804, E-mail: kly@biomed.au.dk

Supplementary Table 1

Test	Sex (F/M)	Measurements	relative in KI to WT	Mean	s.e.m.	Statistical tests	Correction and/or *post hoc* test	P-values	Significance	Figure
Western blotting	F/M	Cortex	α1	1,235	0,04	T-test		0,0227	*	Fig. 2c
			α2	0,965	0,03	T-test		0,9914		
			α3	0,795	0,008	T-test		< 0.0001	****	
		Hippocampus	α1	1,09	0,07	T-test		0,0393	*	
			α2	1,01	0,06	T-test		0,7441		
			α3	0,86	0,03	T-test		< 0.0001	****	
		Cerebellum	α1	1,38	0,11	T-test		0,0052	**	
			α2	0,99	0,008	T-test		0,9802		
			α3	0,81	0,03	T-test		< 0.0001	****	
		Brain	α1	1,32	0,04	T-test		0,0007	***	
			α2	1,03	0,03	T-test		> 0.9999		
			α3	0,85	0,02	T-test		< 0.0001	****	

Test	Sex (F/M)	Measurements	Genotype (n)	Mean	s.e.m.	Statistical tests	Correction and/or *post hoc* test	P-values	Significance	Figure
Open field	F/M	Accumulated distance	WT=14	72,35	3,395	Unpaired t test	Welch's correction	< 0,0001	****	Fig. 3a
			KI=15	147,3	5,971					
	F/M	Peripheral time	WT= 14	502,1	27,27	Unpaired t test	Welch's correction	0,0043	**	Fig. 3c
			KI=15	638,1	33,91					
	F/M	Meander (Rotation per meter)	WT= 14	88154	7690	Unpaired t test	Welch's correction	0,0002	***	Fig. 3d
			KI=15	49591	2109					
Elevated plus Maze	F/M	Open arm entries	WT= 14	32,58	2,368	Unpaired t test	Welch's correction	< 0,0001	****	Fig. 3f
			KI=15	51,77	2,013					
	F/M	Open arm time	WT= 14	8,779	1,234	Unpaired t test	Welch's correction	< 0,0001	****	Fig.3g
			KI=15	30,01	1,855					
PTZ-indiced epileptic seizures	F/M	Survival curve	WT=9			Unpaired t test	Mann Whitney test	<0.005	*	Fig. 4
			KI=8							
Evoked action potentials	M	RMP (mV)	WT = 13	-66,8	0,8					
			KI = 11	-68,4	1,2					
	M	Rin (MΩ)	WT = 13	39,1	2,5					
			KI = 11	36,4	4,3					
	M	Action potential	WT = 13	-54,2	0,8					
			KI = 11	-55,3	0,6					

	M	Amplitude	WT = 13	81,2	1	Unpaired t-test				Fig.5a
			KI = 11	83,3	1,1					
	M	Half-width	WT = 13	0,79	0,03					Fig.5a
			KI = 11	0,85	0,02					
	M	Rate of rise	WT = 13	386,7	12,2					Fig.5a
			KI = 11	397,9	15,9					
	M	Rate of decay	WT = 13	103,9	2,9			0,040	*	Fig.5a
			KI = 11	95,4	2,7					
	M	Depolarizing current pulses	WT = 13			Unpaired t-test				Fig. 5b
			KI = 11							
	M	Variance of inter-spike intervals	WT = 8	1558,7	797,1	Unpaired t-test	Mann-Whitney Rank Sum test	0.007		Fig. 5c
			KI = 8	531,6	235,3					
	M	Steady-state electroresponsive	WT = 8				two way ANOVA	0,42		Fig. 5d
			KI = 8							
	M	Activity-dependent reduction	WT = 12	32	3	Unpaired t-test		0,2		Fig. 5e
		(%)	KI = 12	26	2					
	M	Afterhyperpolarization	WT = 12	9,1	0,9	Unpaired t-test		0,12		Not shown
		(AHPs)	KI = 12	7,2	0,6					
Barnes Maze	F	Total latency	WT= 8			two way ANOVA	Sidak Multiple comparisons test	0,0348	*	Fig. 6a
			KI=6							
	F	Primary latency	WT= 8			two way ANOVA	Sidak Multiple comparisons test	0,2007	ns	Fig. 6b
			KI=6							
	F	Number of visits to target	WT= 8			two way ANOVA	Sidak Multiple comparisons test	0,0081	**	Fig. 6c
			KI=6							
	F	Total path length	WT= 8			two way ANOVA	Sidak Multiple comparisons test	0,0263	*	Not shown
			KI=6							
		Mean speed	WT= 8			two way ANOVA	Sidak Multiple comparisons test	0,9222	ns	Not shown
			KI=6							
Passive avoidance	F/M	WT (Veh) vs α_3^{D801Y} (Veh)	WT= 8	260,8	44,63	Unpaired t-test		0,0136	*	Fig. 6f
			KI=6	7,6	4,402					
		WT (Veh) vs α_3^{D801Y} (Clz)	WT= 8	260,8	44,63	Unpaired t-test		0,6437	ns	Fig. 6f
			KI=6	351,1	94,9					
		α_3^{D801Y} (Veh) vs. α_3^{D801Y} (Clz)	KI (veh)=4	7,6	4,402	Unpaired t-test		0,0201	*	Fig. 6f
			KI (Clz)=6	351,1	94,9					
		ΩT (Clz) vs. α_3^{D801Y} (Clz)	WT (Clz)=4	227,9	65,2	Unpaired t-test		0,3687	ns	Fig. 6f
			KI (Clz)=6	351,1	94,9					
Hippocampla granule cells	F	DG granule cell number	WT=10	477124	25442					
			KI=10	408217	16500	Unpaired t test	Welch's correction	0,0378	*	Fig. 7b

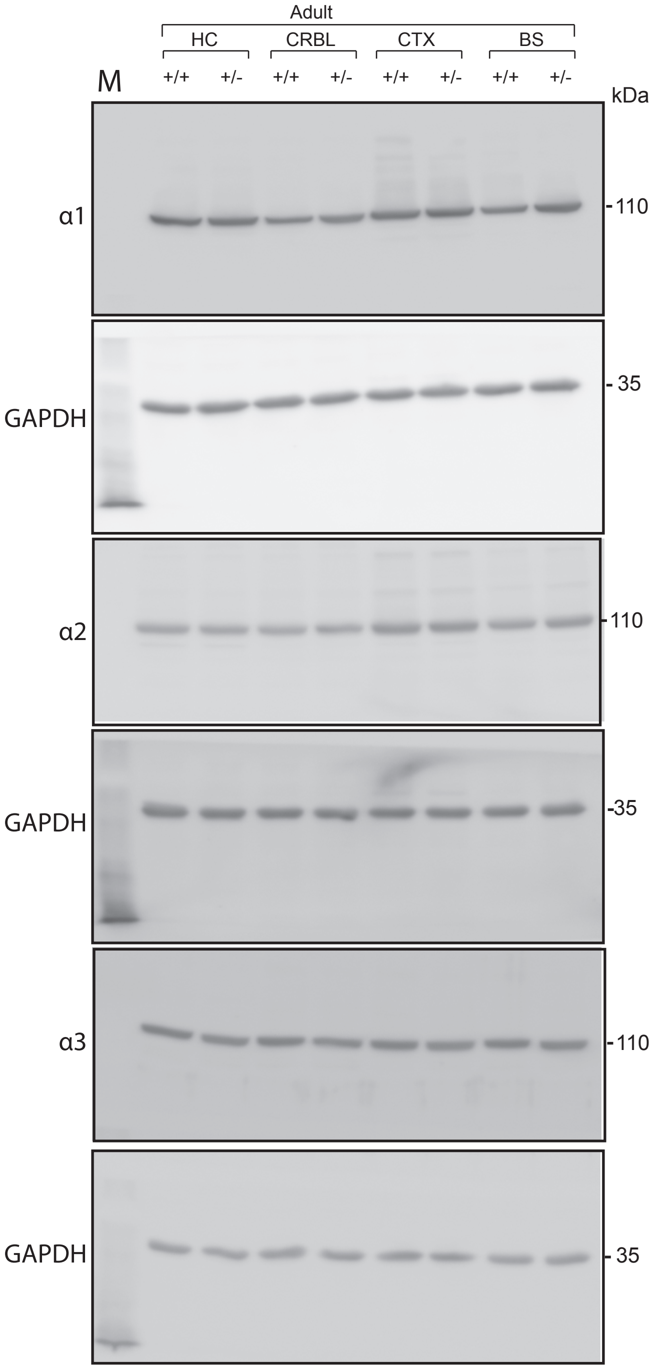

Supplementary Figure 1. Full view Western blots illustrating α1-3 protein expression in α3+/D801Y and WT mice in cortex (CTX), hippocampus (HC), cerebellum (CRBL) and in whole brain lysates (Brain). M denotes Marker lane.
